# Phospholipid scramblase 1 (PLSCR1) is a novel substrate of NEDD4-2 (NEDD4L) mediated ubiquitination

**DOI:** 10.1038/s41420-025-02700-9

**Published:** 2025-08-20

**Authors:** Meriam Shabbar, Jantina A. Manning, Yoon Lim, Sonia S. Shah, Diva Sinha, Andrej Nikolic, Jarrod J. Sandow, Sharad Kumar

**Affiliations:** 1 Centre for Cancer Biology, University of South Australia, Adelaide, SA Australia; 2 Adelaide University, Adelaide, SA Australia; 3 The Walter and Eliza Hall Institute of Medical Research, Parkville, VIC Australia; 4 Department of Medical Biology, University of Melbourne, Parkville, VIC Australia; 5 Adelaide Medical School, The University of Adelaide, Adelaide, SA Australia; 6 Present Address: IonOpticks, Collingwood, VIC Australia

**Keywords:** Cell death, Cell signalling

## Abstract

NEDD4-2 (human NEDD4L), a ubiquitin ligase, plays an essential role in regulating a number of membrane proteins, including ion channels and transporters. In the kidney, NEDD4-2 deletion results in a progressive loss of tubular cells and salt-sensitive chronic kidney disease. While deregulation of sodium homeostasis due to increased levels and function of the epithelial sodium channel (ENaC) and sodium chloride transporter (NCC), both NEDD4-2 substrates, plays a critical role in kidney damage in this model, other ubiquitination targets may also be important. Here, we employed an affinity purification mass spectrometry approach to identify additional interactors of NEDD4-2 in kidney cells and discovered phospholipid scramblase 1 (PLSCR1) as a new NEDD4-2 substrate. We show that PLSCR1 is a direct interactor and substrate of NEDD4-2. As a result, NEDD4-2 deficiency both in cultured cells and in mouse kidney resulted in increased levels of PLSCR1 protein. We observed increased phosphatidyl serine exposure in NEDD4-2 knockout cells in response to both calcium and apoptotic stimuli and this phenotype was reversed when NEDD4-2 expression was restored. Consistently, apoptotic cells lacking NEDD4-2 showed a higher rate of macrophage clearance. Together, these results indicate that PLSCR1 is a novel substrate of NEDD4-2-mediated ubiquitination and that NEDD4-2 regulates PLSCR1 protein stability and function.

## Introduction

NEDD4-2 is a member of the NEDD4 family of HECT-type E3 ubiquitin ligases implicated in the regulation of a large number of membrane proteins [1–3]. In the kidney, NEDD4-2 is a major contributor to kidney health through the regulation of multiple ion channels and transporters [3, 4]. Our previous studies have demonstrated that kidney tubule-specific deletion of *Nedd4-2* in mouse results in progressive kidney damage with similarities to human chronic kidney disease (CKD), characterised by the loss of tubular epithelia, immune cell infiltration, interstitial fibrosis and loss of kidney function [5, 6]. The kidney damage and loss of renal function are dramatically enhanced when *Nedd4-2* deficient mice are fed a high-sodium diet [7]. Apoptosis accompanies tubular cell loss, however, it remains unknown how the increased Na^+^ reabsorption in *Nedd4-2* deficient mice initiates cell death.

PLSCR1 is a multifunctional transmembrane protein involved in multiple cellular processes, including cell proliferation, cell signalling, transcriptional regulation, viral pathology and apoptosis [8–10]. However, the canonical function of PLSCR1 is its enzymatic activity as a phospholipid scramblase involved in calcium-mediated translocation of phosphatidyl serine (PS) to the outer leaflet of the plasma membrane [11]. PS exposure on the cell surface is an established marker for apoptosis and an important feature of apoptotic cell death, as externalised PS acts as a signal for macrophage clearance [10, 12]. Although PLSCR1 was the first enzyme identified for its scramblase function, its activity appears to be redundant with TMEM16F, another phospholipid scramblase that plays a more critical role in PS externalisation [13, 14]. However, PLSCR1 has also been implicated in other nonredundant cellular functions, including in defence against viral infections, as well as in neuronal and retinal cell death [10, 12, 15].

In this study, we aimed to identify additional substrates for NEDD4-2 in the kidney to fully explore the CKD-like pathology observed in NEDD4-2 deficient animals. Here, using NEDD4-2 as a bait in an affinity purification mass spectrometry approach, we identified PLSCR1 as a new target for NEDD4-2-mediated ubiquitination in kidney cells. We show that NEDD4-2-dependent regulation controls PLSCR1 protein stability and abundance in both cultured kidney cells and mouse kidney tissues. We also demonstrate that NEDD4-2 deficiency results in increased PS exposure following calcium and apoptotic signalling, as well as a higher rate of macrophage clearance of cells.

## Results

### PLSCR1 as a new candidate substrate of NEDD4-2

**Fig. 1:**
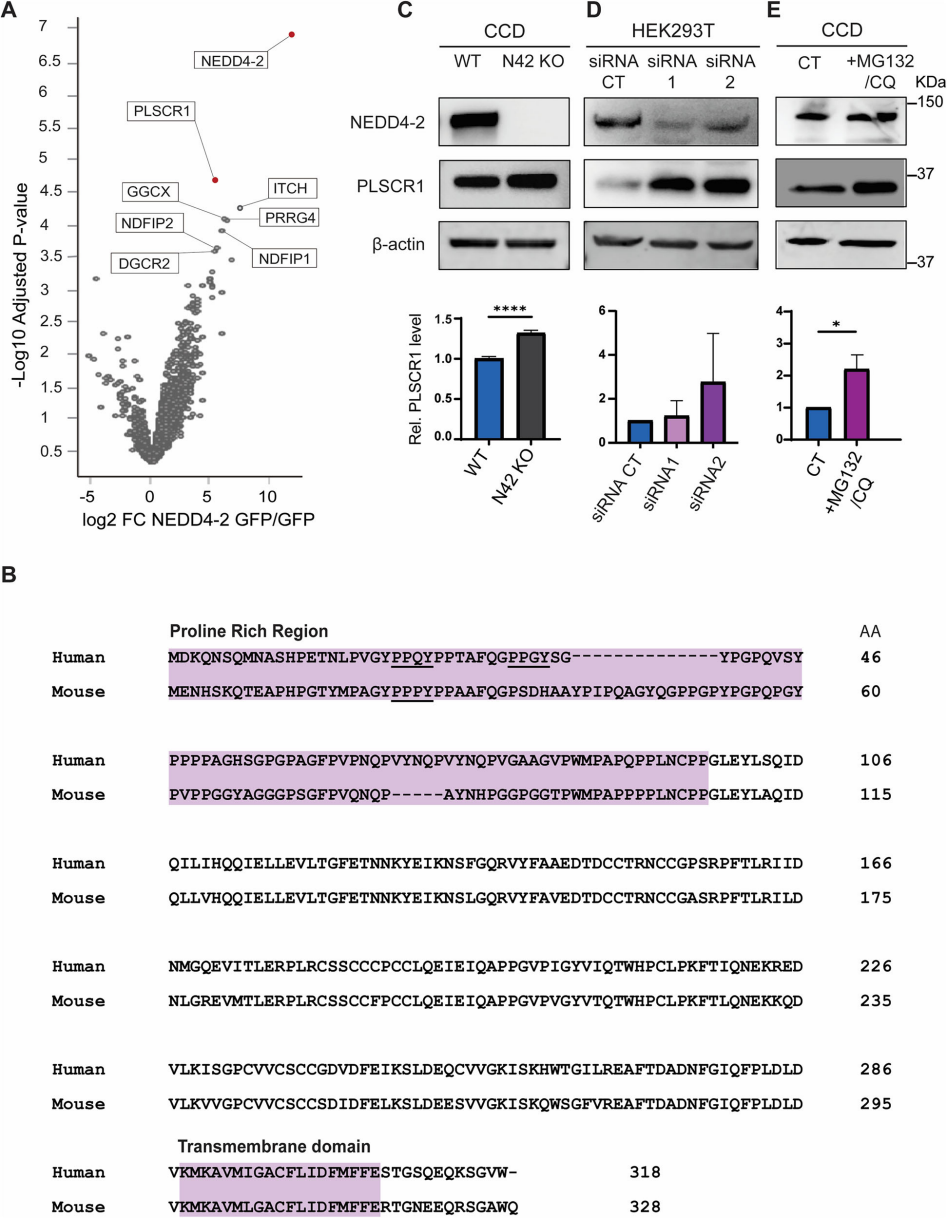
Identifying PLSCR1 as a potential target of NEDD4-2. **A** Proteomic enrichment analysis shows phospholipid scramblase 1 (PLSCR1) as the most enriched protein in the pulldown enrichment dataset (Adj. *p*-value of 0.03) after NEDD4-2, both highlighted in red. **B** Sequence alignment (CLUSTAL-Omega (EMBL-EBI)) of PLSCR1 for human and mouse homologues: *Homo sapiens* PLSCR1 (O15162) and *Mus musculus* PLSCR1 (Q9JJ00). Protein domains that suggest features of NEDD4-2 interacting proteins are highlighted and the PPxY motifs are underlined. Immunoblotting showing increased PLSCR1 protein level in **C** NEDD4-2 KO CCD kidney cells (N42 KO), **D** NEDD4-2 siRNA treated HEK293T cells (siRNA1, 2) and (**E**) after the inhibition of proteasomal and lysosomal degradation by MG132 and chloroquine (CQ) treatment respectively in CCD cells compared to untreated controls (CT); *n* = 3 ± SD, significance was determined using unpaired two-tailed Student’s *t-test*, *p < 0.05, **p < 0.005, ***p < 0.0005, ****p < 0.0001.

To identify new NEDD4-2-interacting proteins in the kidney, enrichment proteomic analysis was performed in a kidney cortical collecting duct cell line (CCD). The interactome analysis revealed PLSCR1 as a highly significant candidate, being the most enriched protein after NEDD4-2 (Fig. 1A). In support of the proteomics finding, the structure of PLSCR1 displays multiple features found in known NEDD4-2 substrates, including a C-terminus transmembrane domain [3] (Fig. 1B). It also contains PY motifs in the cytoplasmic N-terminal, which are known to bind to WW domains (four are found within NEDD4-2) [3]. NEDD4-2 specifically targets PPxY or LPxY motifs for direct PY-mediated interactions with its substrates. In its proline-rich region, highlighted in purple, PLSCR1 has two PPxY motifs in the human homologue and one in the mouse protein (Fig. 1B).

NEDD4-2 substrates are usually targeted for degradation or recycling by the proteasome or lysosome systems, hence, we evaluated the impact of the loss of NEDD4-2 on PLSCR1 levels in mouse NEDD4-2 KO cells and human cells following NEDD4-2 knockdown. NEDD4-2 KO was generated in a mouse CCD cell line previously and displays complete loss of NEDD4-2 protein [6]. NEDD4-2 KO cells showed higher protein levels of PLSCR1 compared to their wild type (WT) CCD counterparts (Fig. 1C). Similarly, knockdown of *NEDD4L* in human HEK293T cells using siRNA revealed a trend towards increased PLSCR1 protein levels (Fig. 1D). In CCD cells, blocking the proteasomal and lysosomal degradation by MG132 and chloroquine (CQ) respectively showed a significant increase in PLSCR1 levels, suggesting its turnover by these pathways (Fig. 1E).

### NEDD4-2 interacts with and ubiquitinates PLSCR1

**Fig. 2:**
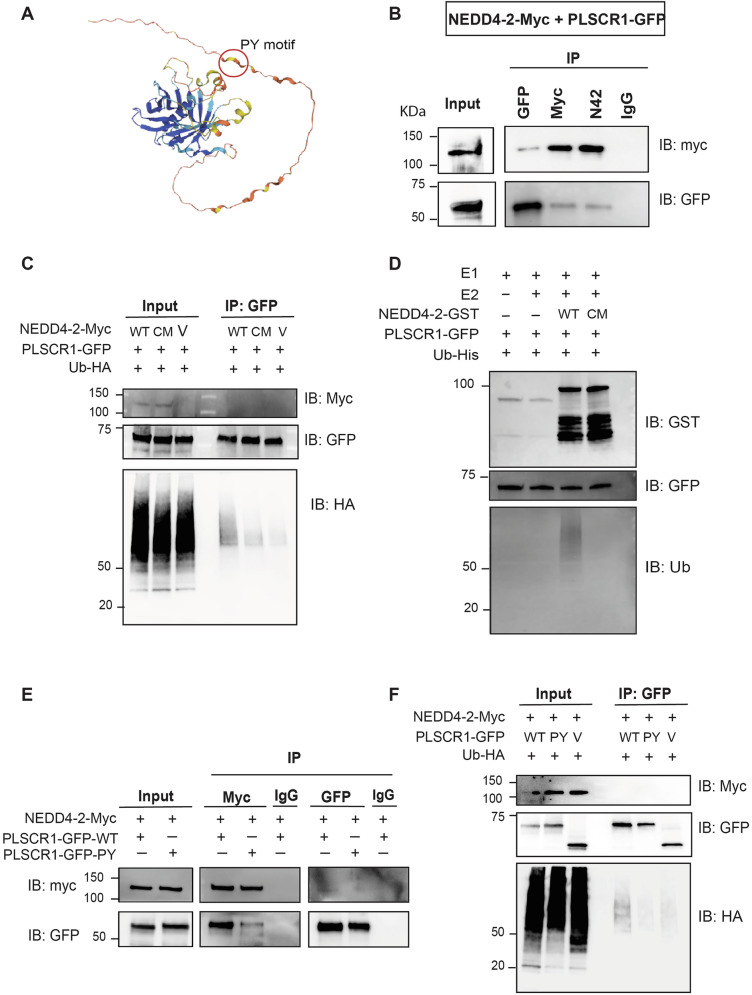
PLSCR1 interacts with and is ubiquitinated by NEDD4-2. **A** The 3D structure of mouse PLSCR1 predicted using alpha fold; highlighted is the N-terminus PY motif (PPPY). HEK293T cells were transfected with PLSCR1-GFP and NEDD4-2-Myc and immunoprecipitation was performed. **B** Co-immunoprecipitation analysis shows a direct interaction between PLSCR1-GFP and NEDD4-2-Myc. **C** HEK293T cells were transfected with PLSCR1-GFP, NEDD4-2-myc and Ub-HA constructs and the GFP tag was immunoprecipitated to check ubiquitination. Ubiquitination assay showing strong ubiquitination of PLSCR1 by WT NEDD4-2 and not by the catalytically inactive Cystine mutant NEDD4-2 (CM) or vector-only control (V). **D** PLSCR1-GFP was incubated with recombinant GST-NEDD4-2, ubiquitin, E1, E2 and ATP for in vitro ubiquitination assay and confirms ubiquitination of PLSCR1 with WT NEDD4-2 only and not with CM NEDD4-2. Immunoprecipitation showing PY mutant PLSCR1 (PLSCR1-GFP-PY) to have reduced NEDD4-2 interaction (**E**) and ubiquitination (**F**) compared to PLSCR1 WT (PLSCR1-GFP-WT).

The structure of mouse PLSCR1 as predicted by AlphaFold demonstrates the accessibility of the PY motif (Fig. 2A). To evaluate whether NEDD4-2 and PLSCR1 interact, co-immunoprecipitation was performed after co-transfection of NEDD4-2-myc and PLSCR1-GFP into HEK293T cells. After immunoprecipitation of NEDD4-2 (using an antibody against either NEDD4-2 or myc), PLSCR1 was also pulled down (Fig. 2B). Similarly, immunoprecipitation of PLSCR1 using GFP antibody resulted in the detection of NEDD4-2, confirming the interaction between NEDD4-2 and PLSCR1 (Fig. 2B).

Next, a ubiquitination assay was performed to assess if the NEDD4-2 interaction with PLSCR1 results in its ubiquitination. Ubiquitin-HA was co-transfected with PLSCR1-GFP and either WT or a Cys mutant (CM) NEDD4-2-myc, which contains a mutation in the HECT domain that renders the protein catalytically inactive [16, 17]. When GFP was immunoprecipitated, a strong ubiquitination tract was detectable with WT, but only at background levels in CM or myc-only controls, confirming the ubiquitination of PLSCR1 by NEDD4-2 (Fig. 2C). PLSCR1 ubiquitination by NEDD4-2 was further tested in an in vitro ubiquitination system using purified proteins of the ubiquitin system, including GST-tagged NEDD4-2, with ubiquitination assessed by GST pulldown (Fig. 2D). A ubiquitination tract was detectable in the presence of WT NEDD4-2 but absent in the presence of catalytically inactive, CM NEDD4-2. These results demonstrate that NEDD4-2 directly interacts with PLSCR1 and promotes its ubiquitination.

To determine if NEDD4-2 interaction is facilitated by the PY motif, PY mutant PLSCR1 was generated, in which Tyr of its only PY motif (PPPY) was replaced with Ala residue. The mutant protein was then used for co-immunoprecipitation and ubiquitination analyses. While NEDD4-2 showed an interaction with both WT and PY mutant PLSCR1, the interaction with the PY mutant was much weaker than WT, suggesting the interaction is at least partly dependent on the PY motif (Fig. 2E), but may also involve other binding regions. Ubiquitination assays were also performed to examine the impact of the PY mutant on PLSCR1 ubiquitination by NEDD4-2. Interestingly, despite the observed partial interaction, the PY mutant showed reduced ubiquitination by NEDD4-2 (Fig. 2F), suggesting that a strong, stable interaction is required for the effective ubiquitination of PLSCR1. Overall, these results confirm interaction and ubiquitination of PLSCR1 by NEDD4-2 in a PY-dependent manner.

### NEDD4-2 regulates PLSCR1 scramblase activity in response to calcium activation

**Fig. 3:**
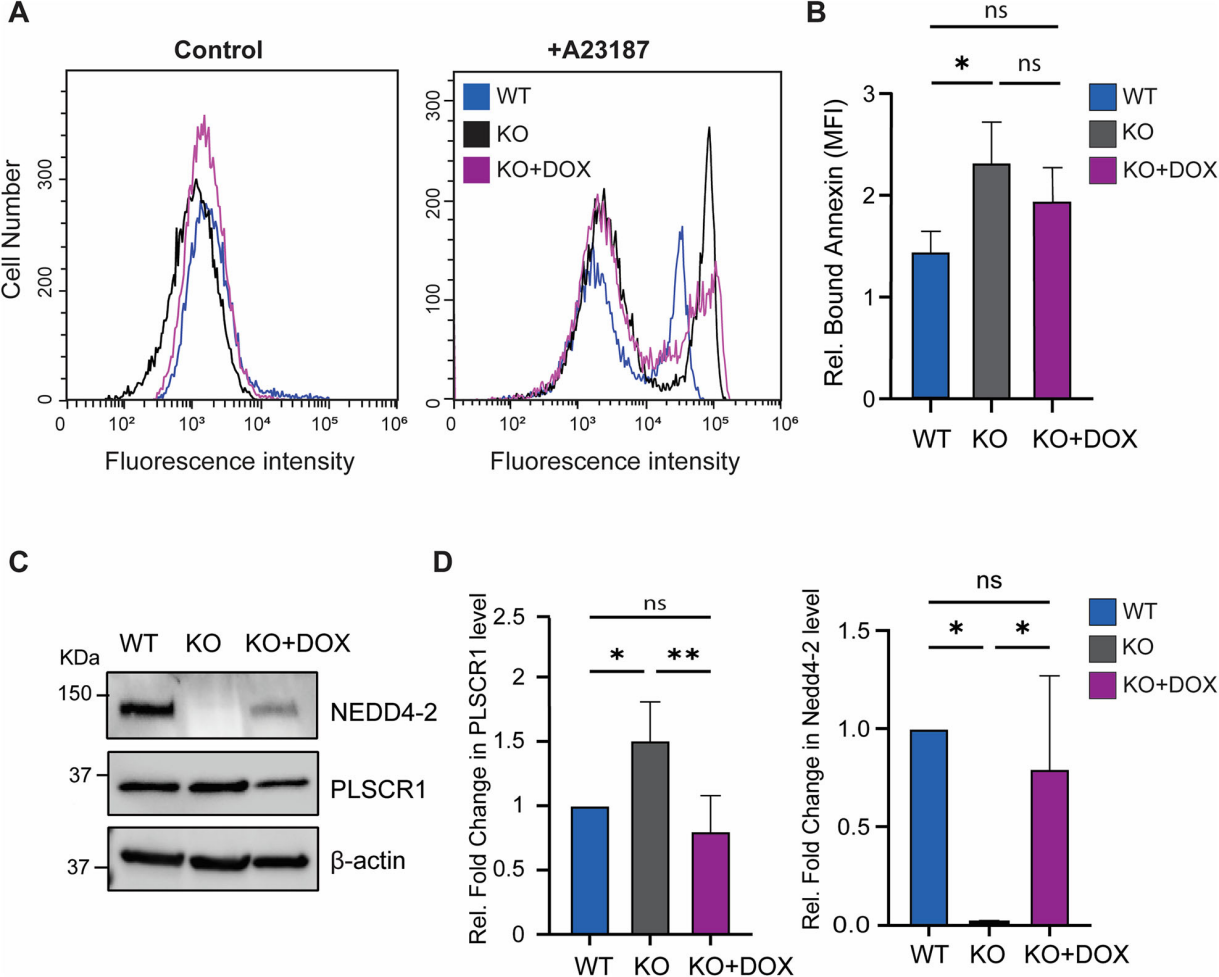
PLSCR1 activation by calcium is influenced by NEDD4-2. NEDD4-2 WT, KO, and doxycycline induced KO (KO + DOX) CCD cells were incubated with calcium ionophore (A23187) and PS exposure was measured by Annexin V. **A** Flow analysis showing the increase in Annexin V fluorescence intensity response to A23187 treatment, highest in Nedd4-2 KO cells (black line). **B** Bound annexin presented as fold change from untreated control, is highest in the NEDD4-2 KO cells and this increase correlates with an increase in PLSCR1 levels as presented by immunoblotting in (**C**) and quantitated in (**D**). MFI represents the GeoMean of fluorescent intensity. PLSCR1 level is measured relative to β-actin and presented as fold change from WT. The flow images are representative of at least three independent experiments. Data presented as mean ± SEM with significance calculated by ordinary one-way ANOVA, ns = non-significant, * *p* < 0.05. **p < 0.005.

To investigate whether the regulation of PLSCR1 protein level by NEDD4-2 impacts its function, we assessed PLSCR1 scrambling activity. PLSCR1 scrambling function is known to be mediated by calcium [18]. Hence, we measured calcium-induced activation of PLSCR1 in WT and inducible NEDD4-2 KO CCD cells. These cells lack NEDD4-2, but its expression can be induced by doxycycline treatment (DOX). WT and NEDD4-2 KO cells were treated with calcium ionophore A23187 and stained with ANNEXIN V to assess PS exposure as a measure of PLSCR1 activity (Fig. 3A). A23187 treatment caused a partial shift in fluorescence intensity, with NEDD4-2 KO cells displaying higher fluorescence when compared to WT as measured by mean fluorescence intensity (MFI) normalised to the basal level of equivalent resting control cells (Fig. 3B). On the other hand, restoring NEDD4-2 expression in the KO cells using DOX treatment reduced the calcium-induced activity of PLSCR1 (Fig. 3A, B). Notably the increased MFI in NEDD4-2 KO cells correlated with higher PLSCR1 levels compared to lower MFI and PSLCR1 levels in WT and NEDD4-2 restored (DOX-induced KO) cells (Fig. 3C). These data reveal that calcium-induced PLSCR1 scrambling activity is impacted directly by NEDD4-2, confirming the involvement of NEDD4-2 in PLSCR1 regulation.

### NEDD4-2 KO results in higher PLSCR1 scrambling activity in response to apoptotic stimuli

**Fig. 4:**
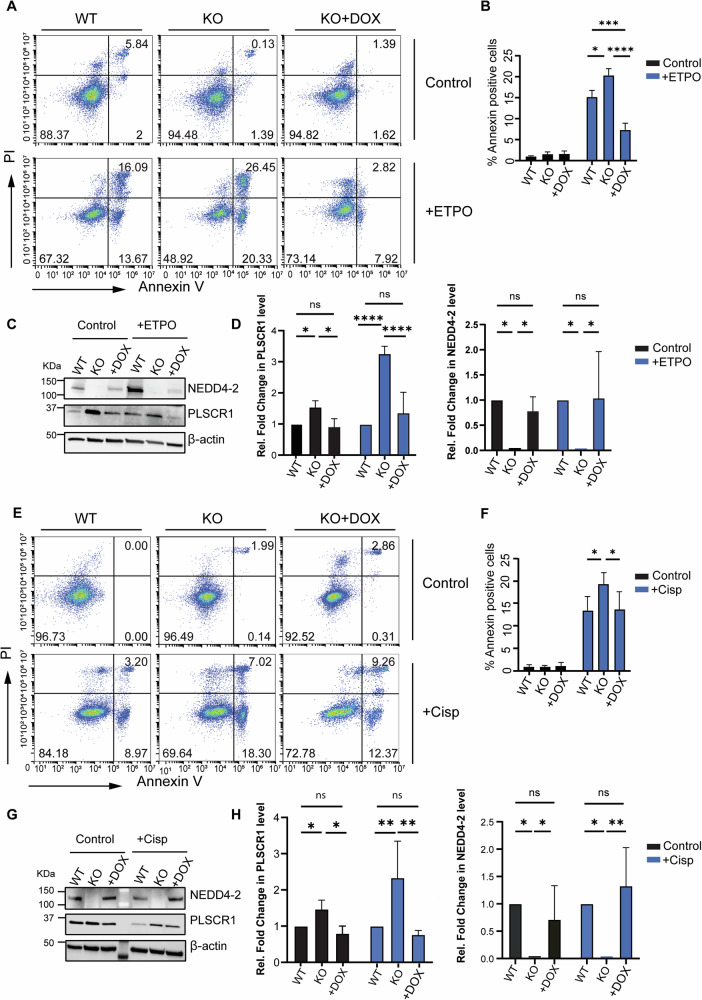
NEDD4-2 level regulates the apoptotic response of cells by regulating PLSCR1 scrambling activity. NEDD4-2 WT, KO and doxycycline induced KO (KO + DOX) CCD cells were treated with Etoposide (ETPO) or Cisplatin (Cisp) for 24 h and PS exposure was measured by Annexin V and PI staining. **A** Flow cytometry analysis showing the Annexin V and PI profile of Etoposide treated cells. **B** PS exposure in response to Etoposide is significantly higher in NEDD4-2 KO cells compared to WT and DOX-treated KO cells. **C** Immunoblotting showing increased PLSCR1 expression in the absence of NEDD4-2. **D** Quantitation of PLSCR1 and NEDD4-2 protein levels relative to β-actin and expressed as a fold change relative to WT. **E** Flow cytometry analysis showing the Annexin V and PI profile of Cisplatin-treated cells. **F** PS exposure in response to Cisplatin is significantly higher in NEDD4-2 KO cells compared to WT and DOX-treated KO cells. **G** Immunoblotting showing increased PLSCR1 expression in the absence of NEDD4-2. **H** Quantitation of PLSCR1 and NEDD4-2 protein levels relative to β-actin and expressed as a fold change relative to WT. The flow images and western blots are representative of at least 3 independent experiments, mean ± SEM for the flow data and mean ± SD for the protein quantification with significance calculated by two-way ANOVA, *p < 0.05, **p < 0.005, ***p < 0.0005, ****p < 0.0001.

PS externalisation is a significant part of the cellular apoptotic response, and PLSCR1 has been reported to be involved in this process [19–21]. Given the involvement of NEDD4-2 in the regulation of PLSCR1, we explored the impact of NEDD4-2 on the cellular apoptotic response through PLSCR1 regulation. Apoptosis was induced in CCD WT and NEDD4-2 KO cells with either etoposide or cisplatin treatment and PS exposure measured as an indication of PLSCR1 apoptotic scrambling activity. WT and KO untreated controls showed no difference in basal PS externalisation (Fig. 4A, D). Apoptotic treatment with either cytotoxic drug resulted in increased scrambling activity in both WT and KO cells (Fig. 4A, D). Importantly, NEDD4-2 KO cells displayed a significantly higher increase in PS exposure after drug treatment than WT cells (Fig. 4B, E) correlating with higher PLSCR1 protein level in these cells (Fig. 4C, F). Notably, this increase in apoptotic response in NEDD4-2 KO cells was reversed upon the re-expression of NEDD4-2 in these cells using DOX (Fig. 4B, E), further supporting the regulation of PLSCR1 by NEDD4-2. Together, these results implicate NEDD4-2 in influencing the apoptotic PS scrambling response through the regulation of PLSCR1.

### Elevated PLSCR1 levels in NEDD4-2 KO cells promote increased phagocytic clearance by macrophages

**Fig. 5:**
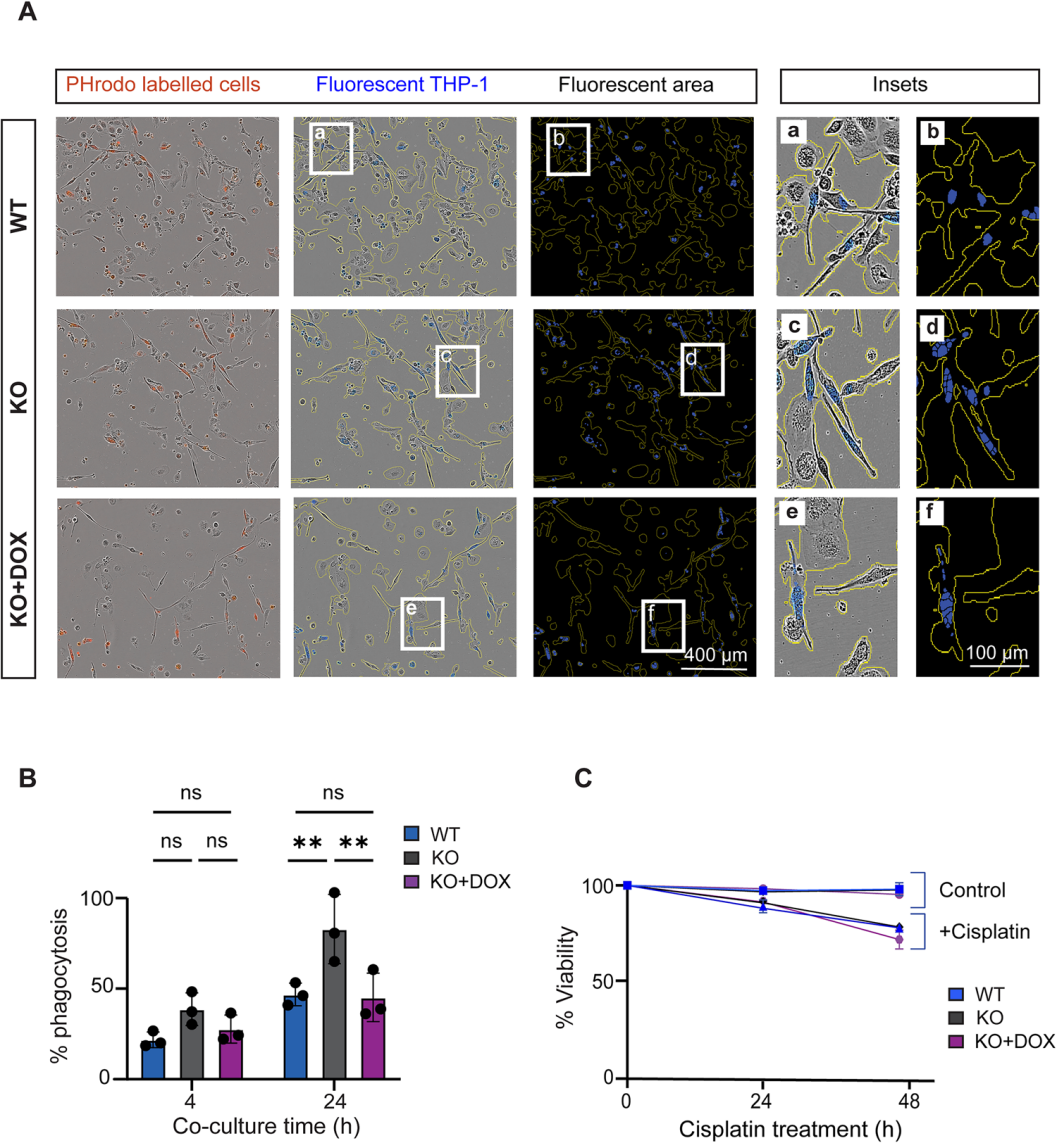
Increased PLSCR1 level in NEDD4-2 KO cells promotes increased phagocytotic clearance by macrophages. Cisplatin-treated WT, NEDD4-2 KO and DOX induced KO CCD cells were co-cultured with THP-1 cells and a pH sensitive dye (pHrodo) was used to measure the rate of macrophage clearance. **A** Representative live cell images showing the rate of phagocytosis of NEDD4-2 KO cells at 24 h. Red fluorescence represents labelled CCD cells successfully engulfed by THP-1. Blue mask applied to fluorescent THP-1 to eliminate background fluorescence. The % of phagocytosis is calculated by the ratio of the integrated fluorescent area highlighted in blue to the total cell area outlined in yellow. **B** Statistical analysis of phagocytic clearance rate showing a significantly higher clearance of NEDD4-2 KO cells after 24 h incubation. **C** Survival assay showing no difference in death rate over time between WT, KO and KO + DOX cells in response to Cisplatin treatment. Scale bar: 400 μm or 100 µm (insets); *n* = 3. Data presented as mean ± SD for engulfment assay and mean ± SEM for survival assay with significance calculated by two-way ANOVA, ns = non-significant, **p < 0.005.

PS externalisation on apoptotic cells promotes phagocytic clearance by macrophages. To investigate the effect of increased PS scrambling in apoptotic NEDD4-2 KO cells on the rate of macrophage clearance, we performed an efferocytosis assay using THP-1 macrophages and tracked cells using live imaging. Cisplatin-treated CCD cells were labelled with pHrodo® Cell labelling dye and co-cultured with differentiated THP-1 cells. The dye is weakly fluorescent under normal pH conditions and becomes increasingly fluorescent under acidic conditions, such as in the lysosomal and proteasomal compartments. Therefore, fluorescent THP-1 cells indicate the successful engulfment of a labelled CCD cell. Hence, the rate of engulfment was measured by the ratio of fluorescent THP-1 cells to the total number of THP-1 cells over time. Initially, there was no difference in the phagocytosis rate at 4 h, however, compared to WT, there was a significant increase in phagocytic clearance of apoptotic NEDD4-2 KO cells after 24 h (Fig. 5A, B). This difference persisted even after 48 h and was consistent across different plated densities of CCD cells (Fig. S1). Importantly, there was no difference in the rate of apoptosis between WT and NEDD4-2 KO cells as shown by survival assays (Fig. 5C). Therefore, the observed difference in phagocytic clearance is likely to be due to the differences in PLSCR1 levels and its PS scrambling activity between these WT and NEDD4-2 KO cells. Consistently, restoring NEDD4-2 expression in the KO cells significantly rescued phagocytic clearance, with levels reduced from KO to WT levels, supporting the impact of NEDD4-2 regulation on PLSCR1 activity in phosphatidylserine externalisation.

### Higher expression of PLSCR1 in the kidney of NEDD4-2 deficient mice (*Nedd4-2*^Ksp1.3^)

**Fig. 6:**
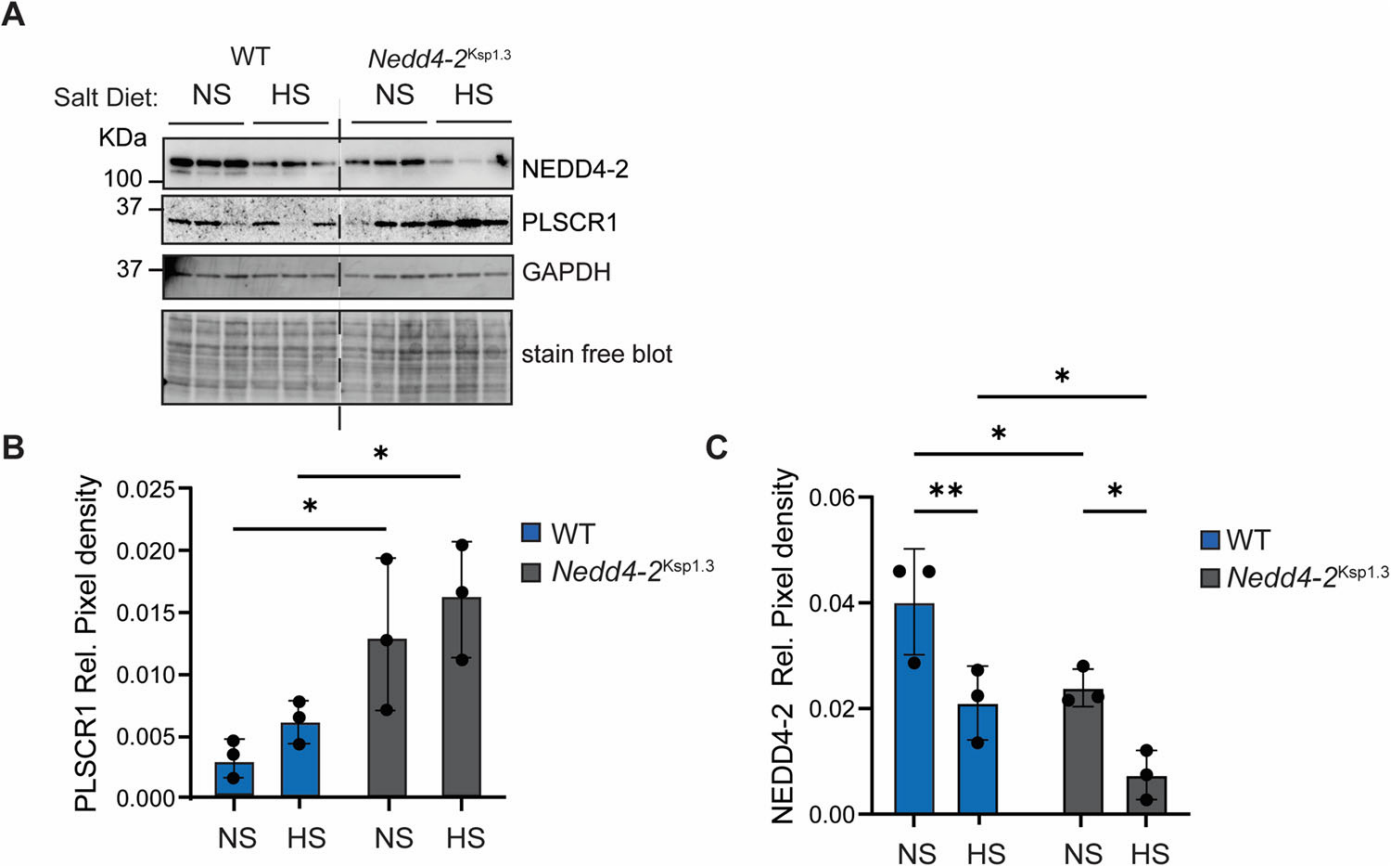
Higher PLSCR1 levels in NEDD4-2 deficient mouse kidney tissues, heightened in the presence of kidney damage. **A** Immunoblot analysis comparing PLSCR1 level between WT and *Nedd4-2*^Ksp1.3^ kidneys after normal salt (NS) or high salt (HS) diets. Quantitation of PLSCR1 **B** and NEDD4-2 **C** expression levels normalised to GAPDH. *n* = 3, mean ± SD, significance was calculated by two-way ANOVA, * *p* < 0.05, **p < 0.005.

To investigate the effect of NEDD4-2 on PLSCR1 in vivo, we analysed kidney tissues from kidney-specific *NEDD4-2* KO mice (*Nedd4-2*^Ksp1.3^), where NEDD4-2 is lost from kidney tubules, most strongly in the cortical collecting duct [5]. Immunoblotting revealed that whole kidney lysates from *Nedd4-2*^Ksp1.3^ mice (which have an approximately 50% reduction in total NEDD4-2 levels), displayed a higher level of PLSCR1 protein compared to WT under normal salt (NS) diet conditions (Fig. 6A, B). The *Nedd4-2*^Ksp1.3^ mice display signs of kidney damage as indicated by multiple markers including kidney injury molecule-1 (KIM-1) and the apoptotic marker cleaved caspase 3 [5]. This damage is exacerbated by high sodium diet [7]. Exposure to high salt (HS) conditions caused a further reduction in NEDD4-2 levels in kidney tissues (Fig. 6C), which correlated with an increasing trend in PLSCR1 levels (Fig. 6B). These results support our in vitro finding that NEDD4-2 regulates PLSCR1 levels.

## Discussion

The ubiquitin-proteasome system (UPS) plays a critical role in kidney homeostasis [22]. Of interest to this study, the ubiquitin ligase NEDD4-2 is critical for renal pathophysiology through the regulation of multiple proteins and signalling pathways [6, 23]. Here, we characterised PLSCR1 as a new interactor and potential substrate of NEDD4-2-mediated ubiquitination.

PLSCR1 is known to undergo several post-translational modifications such as phosphorylation and palmitoylation, which affect PLSCR1 molecular interactions and localisation, respectively [24, 25]. However, the ubiquitination of PLSCR1 has not been reported. In this study, we first established that PLSCR1 levels are impacted by the proteasome and lysosome system as blocking these pathways caused an increase in PLSCR1 levels. This suggests the proteolytic degradation of PLSCR1 by the ubiquitin system. Secondly, we demonstrated that PLSCR1 can bind NEDD4-2 and undergo NEDD4-2-mediated ubiquitination, which consequently influences its abundance. Similar to other NEDD4-2 substrates, PLSCR1 levels are increased in the absence of NEDD4-2, potentially due to diminished ubiquitin-induced degradation by the proteasome and lysosome resulting in its accumulation.

Amongst the many roles of PLSCR1, it is involved in the process of phospholipid scrambling [26]. PS is an important apoptotic marker, normally confined to the inner leaflet of the plasma membrane. During apoptosis, PS is translocated to the outer leaflet of the membrane by scramblases [27]. Therefore, PS exposure is used as a measure of PLSCR1 activity. Interestingly, there was no significant difference in PS exposure between WT and NEDD4-2 KO cells under basal conditions, despite an increase in PLSCR1 expression in NEDD4-2 KO cells. This could be due to the lack of activation signal for PLSCR1 such as calcium activation, given that PLSCR1 is a calcium induced scramblase. PLSCR1 structure contains a Ca^2+^-binding motif that causes a conformational change in PLSCR1 upon binding to calcium, which is believed to enhance its scrambling activity [28]. In addition, Ca^2+^ has been shown to increase PLSCR1 scrambling activity in a dose-dependent manner [18]. Consistently, our results showed that PLSCR1 activity was induced in response to Ca^2+^, and this response was significantly higher in the NEDD4-2 KO cells, likely due to the higher PLSCR1 protein level. Restoring NEDD4-2 expression in these cells reduced PLSCR1 activity. Importantly, even low expression of NEDD4-2 resulted in a measurable restoration in PLSCR1 level and activity, further confirming the regulatory impact of NEDD4-2 on PLSCR1 (Fig. 3).

While PS externalisation is a well-studied downstream effect of caspase activation, the effect of caspases on PLSCR1 scramblase activity is controversial [29, 30]. Our results show that in CCD cells, apoptotic drugs result in increased PLSCR1 scramblase activity as measured by PS exposure. PLSCR1 is not cleaved by caspases, therefore unlike some scramblases that are directly activated by caspase cleavage, the observed activation of PLSCR1 scrambling function is likely to be indirect, potentially due to the Ca^2+^ influx associated with apoptosis or the reported caspase-mediated Protein kinase C – δ (PKC δ) activation of PLSCR1 [20]. Notably, the apoptotic-induced increase in PLSCR1 activity was elevated in NEDD4-2 KO cells, supporting the role of NEDD4-2 in the regulation of PLSCR1. This indicates that NEDD4-2 KO cells are more responsive to apoptotic stimuli possibly due to higher PLSCR1 expression. While these results differ from previous work that showed no correlation between PLSCR1 expression level and PS exposure in response to apoptotic stimuli in cancer cells, this correlation might be cell-type specific as was concluded for PS exposure in that same study [30]. In addition, recent studies have shown tumour cells have highly dysregulated PS signalling due to altered flippase activity and cellular calcium environment, suggesting that these cells might not provide an accurate reflection of PLSCR1 activity [31, 32].

Consistently, increased PLSCR1 levels and apoptotic activity in NEDD4-2 KO cells resulted in enhanced macrophage clearance of these cells. This could suggest a role for NEDD4-2, or the lack thereof, in promoting effective clearance of dying cells, especially in the context of kidney disease, which in turn is associated with low NEDD4-2 levels [5, 33]. However, the difference in macrophage clearance was not evident in the first few hours of coculture, possibly due to the initial low death percentage at 4 hours (Fig. 5B). As the number increased over the following 24 hours to 25%, the difference in engulfment rate became more distinct. Overall, while PLSCR1 is often considered a weak scramblase and its activity potentially masked by other scramblases, our results show that changes in PLSCR1 levels resulted in a measurable difference in PS externalisation both in response to calcium activation and apoptosis. Increased PLSCR1 also correlated with increased macrophage clearance, further supporting the significance of PLSCR1 in PS presentation in these cells. Therefore, our results suggest that the physiological significance of PLSCR1 scrambling activity could be cell-type specific and is influenced by post-translational regulation. Finally, the regulation of PLSCR1 levels by NEDD4-2 is consistent with our in vivo data, where lower NEDD4-2 correlated with significantly higher PLSCR1 levels in the kidney tissue of kidney-specific KO mice (*Nedd4-2*^Ksp1.3^).

Collectively, our results confirm that PLSCR1 is a novel substrate regulated by NEDD4-2. Given the diverse functions associated with PLSCR1, including cell signalling and viral response, it will be interesting to explore the significance of this regulatory mechanism in these additional contexts.

## Materials and methods

### Animal models

*Nedd4-2*^Ksp1.3^ (C57BL/6 J) mice were generated in our laboratory as previously described [5]. All animal studies were approved by the institutional animal ethics and biosafety committees of the University of South Australia and were carried out according to the National Health and Medical Research Council guidelines. Kidney tissues were collected from the salt diet study as described previously [6]. Briefly, mice aged 6–8 weeks were fed either standard sodium chow (0.2% Na^+^) or high sodium chow (3.1% Na^+^) for 17 days. For kidney collection, animals were humanely killed by cervical dislocation and the kidneys were removed and dissected out of the fibrous capsule. The kidneys were first lysed in ice-cold extraction buffer at pH 7.5 (50 mM Tris-HCl, pH 7.5, 1 mM EDTA, 1 mM EGTA, 0.27 M sucrose, 0.1% *β*-mercaptothion and HALT protease and phosphatase inhibitor cocktail; Thermo Fisher Scientific). Lysed tissue was then homogenised by freeze-thaw in liquid nitrogen and incubated on a nutator for 30 min at 4 °C. Protein supernatant was separated by centrifugation at 13000 rpm for 5 min. 25 μg of protein was combined with protein loading buffer (100 mM Tris-HCl pH 6.8, 200 mM DTT, 4% SDS, 0.2% bromophenol blue, 20% glycerol) and boiled at 95 °C for 5 min. Immunoblotting was then performed as described below. For histological analysis, kidneys were transferred to 70% ethanol and then embedded in paraffin.

### Cell culture

Mouse cortical collecting cell line mCCD-N21 was grown in DMEM/F12 media (Sigma-Aldrich) supplemented with 2% foetal bovine serum (FBS, JHR Biosciences), 1% Insulin-Transferrin-Selenium-Ethanolamine (ITS), 1 nM 3,3′,5-triiodo-L-thyronin, 10 ng/mL Epidermal growth factor (EGF) and 50 nM dexamethasone (all from Sigma-Aldrich). Cells were maintained at 37 °C and 5% CO^2^ atmosphere. *NEDD4-2* knockout (KO) CCD cell line was generated using CRISPR–Cas9 as described previously and validated by immunoblotting using NEDD4-2 antibody [6]. HEK293T cells were grown in DMEM media (Sigma-Aldrich) supplemented with 10% FBS and were cultured in the same conditions as CCD cells. THP-1 cells were grown in RPMI (Sigma-Aldrich) media supplemented with 10% FBS and were cultured in the same conditions as CCD cells.

### Immunoblotting

Cells were lysed in RIPA buffer (10 mM Tris-Cl, 150 mM NaCl, 0.1% SDS, 1% NP-40, 1% deoxycholate pH 7.5), frozen and then thawed on a nutator for 30 min at 4 °C and centrifuged at 13,000 rpm for 5 min. 25 μg of supernatants were mixed with protein loading buffer and boiled at 95 °C for 5 min. The lysate was then loaded onto 4-20% precast sodium dodecyl sulphate-polyacrylamide gels (Bio-Rad) and transferred to PVDF membrane using the Trans-blot Turbo instrument (Bio-Rad). Membranes were blocked with 5% skim milk in TBS-T for 30 min and incubated in the following primary antibodies overnight: α-PLSCR1 (Abclonal A3430; 1:1000) or α-NEDD4-2 [34]. Membranes were then incubated with horseradish peroxidase secondary antibodies (Millipore) in 5% skim milk in TBS-T for 2 h and developed using ECL Prime (GE Healthcare) or West Femto (Thermo Scientific) on a ChemiDoc Touch Imager (BioRad) and quantitated using Image Lab software (BioRad). All blots were re-incubated with *β*-actin (Sigma AC-15, 1:2000) and developed using either ECL or Cy5 secondary antibody (GE Healthcare). Protein levels were normalised to *β*-actin.

### siRNA-mediated knockdown

HEK293T cells were seeded in a 6-well plate at a density of 2 × 10^5^ for 24 h. 20 nM of control or NEDD4-2 siRNA was diluted in OptiMEM (Gibco) and mixed with 9 uL of Lipofectamine RNAiMAX (Invitrogen) according to the manufacturer’s instructions. Treated cells were incubated for 48 h in normal cell culture conditions, as described above. Cells were then collected by trypsinisation and were processed for immunoblotting as above. Control siRNA was purchased from GenePharma (5’- UUCUCCGAACGUGUCACGUTT-3’) and NEDD4-2 siRNA purchased from Sigma Aldrich: siRNA1 (5’-AACCACAACACAAAGUCACACTT-3’), siRNA2 (5’- GGAGACAGCAUUCUAUUUATT-3’).

### NEDD4-2 construct and GFP-Trap immunoprecipitation

The short isoform of mouse *Nedd4-2* (lacking the C2 domain: XP_036017205.1) was cloned from mouse kidney cDNA into pEGFP-N1 to generate a C-terminal GFP tag. To transfect cells for proteomic analysis, 1.5 × 10^6^ cells were seeded per 100 mm dish and transfected the following day with 10 μg mNedd4-2-GFP or pEGFP-N1 as a control using Lipofectamine 3000 reagent as per manufacturer’s instructions (Thermo Fisher). Eight dishes per transfected group were used per experiment, with the experiment being repeated in triplicate. 24 h after transfection, 20 μM MG132 (Sigma Aldrich) and 20 μM CQ (Sigma Aldrich) were added to the media for 4 h to block the proteasomal and lysosomal degradation, respectively. Cells were detached using trypsin (Gibco) and pooled, resuspended and washed three times in ice-cold 1xPBS. Cells were lysed in lysis buffer comprising 150 mM NaCl, 20 mM Tris, pH 7.5, 2 mM EDTA, 10% glycerol and 1% Triton X-100 supplemented with protease and phosphatase inhibitors and 50 µM PR-619. Following the extraction of soluble proteins, GFP-tagged proteins were bound to GFP-Trap Magnetic Agarose beads (Chromotek) and isolated following manufacturers protocols. Briefly, lysates were bound to washed beads and incubated at 4 °C for 3 h. Following washing, GFP-tagged proteins were eluted in 0.2 M glycine pH 2.5 before the addition of neutralisation buffer, 1 M Tris pH 10.4. Five μl was subject to immunoblotting with primary rabbit α-GFP (Abcam ab260, 1:1000) overnight at 4 °C. After washing in PBST, membranes were incubated for 2 h at RT with secondary conjugated Horseradish Peroxidase (HRP) (GE Healthcare). HRP signals were detected using ECL Plus (GE Healthcare), developed on a ChemiDoc MP Imager (BioRad) and analysed by Image Lab software (BioRad). Remaining eluates were processed for proteomic analysis.

### Mass spectrometry and proteomic analysis

Three biological replicates were analysed for each sample. Protein samples were resuspended in 6 M Urea, 100 mM DTT and 100 mM Tris-HCl pH 7.0 and subjected to protein digestion using FASP (filter-aided sample preparation) before lyophilisation to dryness using a SpeedVac AES 1010 (Savant, Thermo Fisher) [35]. Peptides were resuspended in 2% acetonitrile, 1% formic acid and injected and separated by reversed-phase liquid chromatography on a M-class UPLC system (Waters) using a 250 mm × 75 µm column (1.6 µm C18, packed emitter tip; IonOpticks, Australia) with a linear 90 min gradient at a flow rate of 400 nl/min from 98% solvent A (0.1% Formic acid in Milli-Q water) to 34% solvent B (0.1% Formic acid, 99.9% acetonitrile). The UPLC was coupled online to a freshly cleaned Q-Exactive HF-X mass spectrometer (Thermo Fisher). The Q-Exactive HF-X was operated in a data-dependent mode, switching automatically between one full-scan and subsequent MS/MS scans of the 15 most abundant peaks. Full-scans (m/z 350–1,600) were acquired with a resolution of 60,000 at 200 m/z. The 15 most intense ions were sequentially isolated with a target value of 100,000 ions and an isolation width of 1.4 m/z and fragmented using HCD with normalised collision energy of 27. Maximum ion accumulation times were set to 50 ms for full MS scan and 40 ms for MS/MS. Dynamic exclusion was enabled and set to 20 s.

Raw files were analysed using MaxQuant software (version 1.5.8.3) and the database search was performed using mouse sequences obtained from Uniprot, including isoforms with strict trypsin specificity allowing up to two missed cleavages. The minimum required peptide length was set to seven amino acids. Carbamidomethylation of cysteine was set as a fixed modification, while N-acetylation of proteins N-termini and oxidation of methionine were set as variable modifications. During the MaxQuant main search, precursor ion mass error tolerance was set to 4.5 ppm and fragment ions were allowed a mass deviation of 20 ppm. PSM and protein identifications were filtered using a target-decoy approach at a false discovery rate (FDR) of 1%. Further analysis was performed using a custom pipeline developed in R, which utilises the LFQ intensity values in the MaxQuant output file proteinGroups.txt. Proteins not found in at least 50% of the replicates in one group were removed. Missing values were imputed using a random normal distribution of values with the mean set at mean of the real distribution of values minus 1.8 s.d., and a s.d. of 0.3 times the s.d. of the distribution of the measured intensities. The probability of differential protein expression between groups was calculated using the Limma R package. Probability values were corrected for multiple testing using the Benjamini-Hochberg method.

### PLSCR1 construct and immunoprecipitation

Mouse PLSCR1 was cloned into pEGFP-C2 expression vector. PY mutant PLSCR1 was generated from WT PLSCR1-GFP using site directed mutagenesis changing the Tyrosine of the PY motif to Alanine. HEK293T cells were co-transfected with 1 μg of NEDD4-2-myc and 1 μg of PLSCR1-GFP using Lipofectamine3000, as above. 24 h post-transfection, cells were treated with 20 μM MG132 (Sigma Aldrich) and 200 μM CQ (Sigma Aldrich) for 4 h then collected and lysed as above. Lysates were precleared for 30 min at 4 °C with protein G-Sepharose (Amersham Biosciences). Precleared samples were incubated with 10 μl of protein G-Sepharose beads and 0.5 μg of either rabbit α-GFP (as above), mouse *α*-myc (Cell Signalling Technology, 9B11), rabbit α-Nedd4-2 [34] or rabbit α-IgG. The protein-bound beads were then washed twice in lysis buffer and once in PBS, boiled at 95 °C for 5 min and run on 10% SDS-PAGE gels for immunoblotting as above.

### Ubiquitination assays

HEK293T cells were co-transfected with 1 μg of either WT or catalytically inactive cysteine mutant (CM) NEDD4-2-myc [16], 1 μg of ubiquitin-HA and 1 μg of PLSCR1-GFP either WT or PY mutant (described above) using Lipofectamine 3000, as above. 24 h post-transfection, cells were treated with 20 μM MG132 (Sigma Aldrich) and 200 μM CQ (Sigma Aldrich) for 4 h then collected and lysed in RIPA buffer supplemented with 5 mM N-ethylmaleimide (Sigma Aldrich) to inhibit protein deubiquitination. Immunoprecipitation was then performed on the samples as above. For in vitro ubiquitination, PLSCR1-GFP was immunoprecipitated with an anti-GFP antibody (Sigma-Aldrich) and Protein G magnetic beads from HEK293T cells that had been transfected with PLSCR1-GFP. Immunoprecipitated PLSCR1-GFP was eluted by adding 30 µL of elution buffer (0.2 M Glycine, pH 2.5), followed by neutralisation with 10 µL of 1 M Tris (pH 10.2). The elution process was repeated three times and samples pooled. 5 µL of the elutes were used for in vitro ubiquitination reaction mixtures, which contained the ubiquitin-activating enzyme E1 (80 ng; Boston Biochem, Cambridge, MA, USA), the ubiquitin-conjugating enzyme E2 (1 μM; UBCH5b; Boston Biochem), Ubiquitin (1 μg/ml, Boston Biochem), and recombinant GST-NEDD4-2WT or GST-NEDD4-2CA (10 µg/ml) [17] in 30 μl of ubiquitin assay buffer (40 mM Tris at pH 7.5, 10 mM MgCl2, and 0.6 mM dithiothreitol) containing 2 mM ATP. The reactions were incubated at 37 °C for 60 minutes and stopped by addition of 2X protein sample buffer. The samples were subjected to immunoblotting with mouse anti-ubiquitin-HRP (Santa Cruz Biotechnology), rabbit anti-GFP (Cell Signaling Technology), and rabbit anti-GST (G7781, Sigma-Aldrich) antibodies.

### Apoptosis assays

CCD cells (5 × 10^5^) were seeded in a 6-well plate for 24 h. Either Etoposide, 130 μM, (Sigma) or Cisplatin, 50 μM, (Calbiochem) was then added for 24 h. Cells were then trypsinised and collected together with the floating cells. Collected cells were pelleted by centrifugation for 3 min at 1000 x g. and cells were used for either Flow Cytometry analysis or survival assay.

### Measuring PS externalisation using flow cytometry

PS exposure was measured by Annexin V (Invitrogen) binding according to the manufacturer protocol. Briefly, treated cell pellets were resuspended in binding buffer (10 mM HEPES/NaOH pH 7.4; 140 nM NaCl, 2.5 mM CaCl2) containing 10-fold diluted annexin V and 2 μg/mL propidium iodide (PI) (Sigma). Cells were incubated for 15 min before being analysed on the flow cytometer CytoFlex (Beckman Coulter). At least 10,000 events were counted for each sample. Annexin V was detected on the 488 nm FITC channel and PI on the 610 nm channel. Necrotic cells were identified as PI-stained cells due to the loss of membrane integrity and were gated out from the analysis.

### Survival assays

Apoptosis was induced as above and cells were collected and assessed for 48 h. Cells were collected in triplicate every 24 h and the death rate was measured using Trypan-Blue labelling under light microscopy.

### Inducing calcium signalling

CCD cells (2×10^5^) were seeded in a 6-well plate for 24 h. Cells were washed in PBS and trypsinised, then pelleted by centrifugation at 1000 rpm for 5 min at room temperature. Cells were then resuspended in binding buffer and stimulated with 3.0 μm A23187 (Sigma Aldrich) for 10 min at room temperature. Annexin V (Invitrogen) was then added for another 10 min and the samples were analysed measuring the mean fluorescent intensity (MFI) of live cells on the flow cytometer CytoFlex as above.

### Engulfment assays

THP-1 cells were seeded at 5000 cells/well in a 96-well plate. To differentiate the cells, 1 μg/mL of phorbol 12-myristate 13-acetate (PMA) (Sigma Aldric) was added at the time of seeding and incubated for 48 h. CCD cells were treated with 50 μM of cisplatin (Calbiochem) for 24 h. Treated cells were trypsinised and collected together with floating cells. Collected CCD cells were then labelled with a pH-sensitive dye (pHrodo® Cell Labelling Dye for Incucyte, Sartorius) according to the manufacturer’s instructions. Briefly cells were washed with pHrodo® Wash Buffer to remove media and cellular debris. Cells were then pelleted by centrifugation and resuspended in pHrodo® Labelling Buffer. 50 μg of pHrodo® Cell Labelling Dye was added for 1 h at 37 °C. Labelled cells were then centrifuged at 1300 rpm for 5 min and resuspended in fresh RPMI media. CCD cells were then added to THP-1 cells at multiple concentrations: 7000, 14,000, and 28,000 cells per well. Incucyte S3 live cell analysis system (Sartorius) was used to measure engulfment rate, as the number of fluorescent THP-1 cells over 24 or 48 h period using 20 x objective and 800 ms acquisition for red channel. For the analysis parameters, a fluorescence threshold of 1 RCU was used and a minimum eccentricity of 0.5 was applied. The rate of engulfment was measured using either Red Object Count (ROC) or integrate red fluorescence normalised to phase confluence.

### Statistical analysis

GraphPad Prism software was used for statistical analysis, version 10 (San Diego, CA, USA). Data represent the mean ± SD of at least three independent experiments. Two-way ANOVA with Bonferroni’s post-test was used for multiple-group analysis, unless stated otherwise, whereas two-tailed Student’s *t* tests were used for two-group analysis. Flow cytometry data are presented as mean ± SEM whereas immunoblotting and engulfment analyses are presented as mean ± SD. A *p* value < 0.05 was considered statistically significant.

## Supplementary information

Figure S1

Uncropped Immunoblots

## Data Availability

The mass spectrometry proteomics data have been deposited to the ProteomeXchange Consortium via the PRIDE partner repository with the dataset identifier PXD063842 [36].
