# Supplementary material for: Phospholipid scramblase 1 (PLSCR1) is a novel substrate of NEDD4-2 (NEDD4L) mediated ubiquitination
Source: Cell Death Discov. 2025 Aug 20;11:393. doi: 10.1038/s41420-025-02700-9 (PMC12368045; doi:10.1038/s41420-025-02700-9)
Supplement: Supplementary file 1 — Figure S1 [file 41420_2025_2700_MOESM1_ESM.pdf]

Figure S1

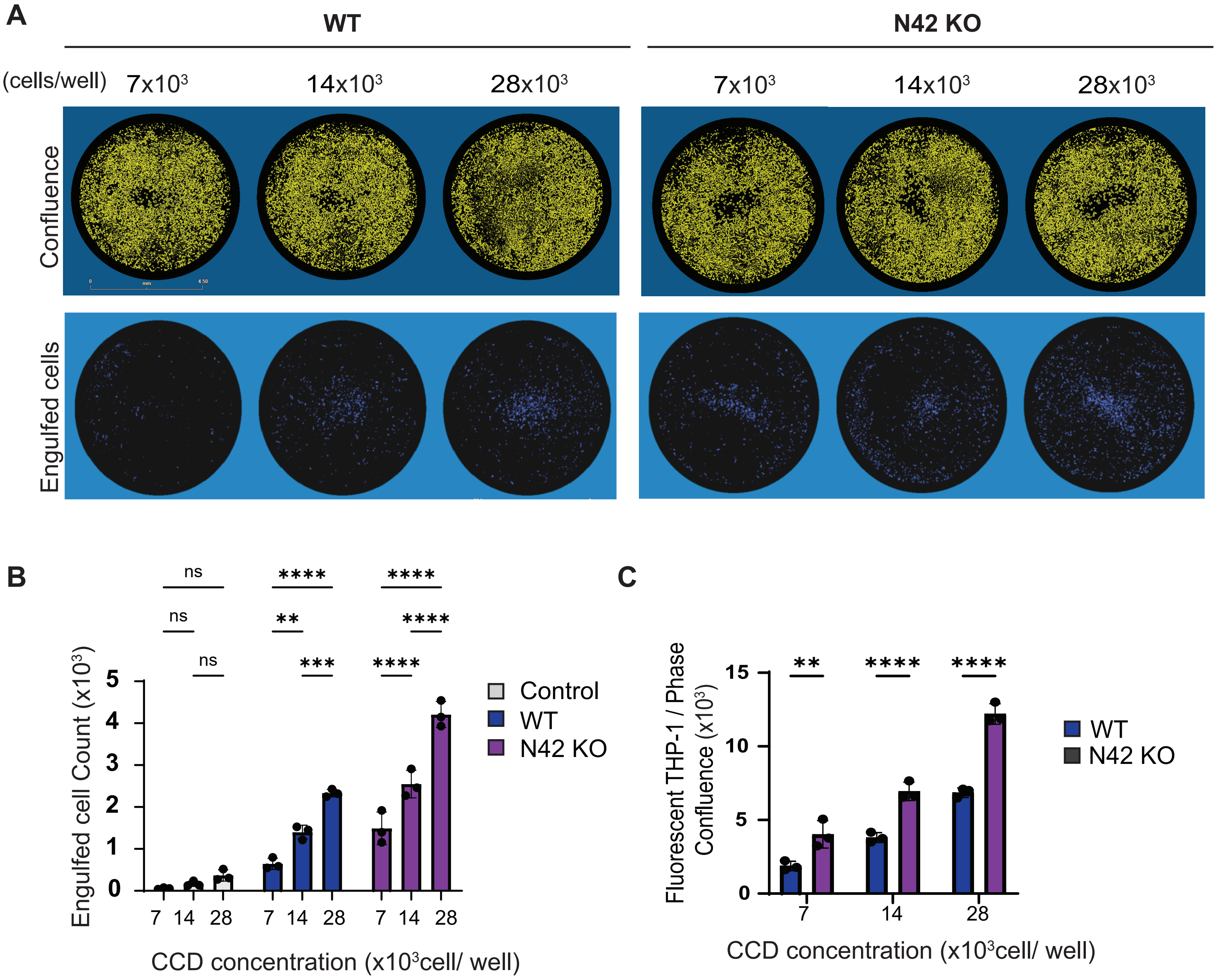

Supplementary Figure 1. The enfulfment rate of apoptotic NEDD4-2 KO cell is pesistently higher than WT. Cisplatin-treated or DMSO-treated (control) CCD cells were co-cultured at different densities with THP-1 macrophages for 48 hours. (A) Whole well images showing cell confluence in yellow and Fluorescent THP-1, which have successfully engulfed CCD cells in blue. Statistical analysis showing the number of engulfed CCD cells by THP-1 in (B) and the rate of engulfment (C) in response to increased concentration of cisplatin-treated WT and NEDD4-2 KO (N42 KO) cells. Scale bar: 4.5 mm, mean ± SEM with significance calculated by 2way ANOVA, ns = non-significant, **p<0.005, ***p<0.0005, ****p<0.0001.
