# Supplementary material for: Phospholipid scramblase 1 (PLSCR1) is a novel substrate of NEDD4-2 (NEDD4L) mediated ubiquitination
Source: Cell Death Discov. 2025 Aug 20;11:393. doi: 10.1038/s41420-025-02700-9 (PMC12368045; doi:10.1038/s41420-025-02700-9)

Figure 1 blots

NEDD4-2

PLSCR1

actin

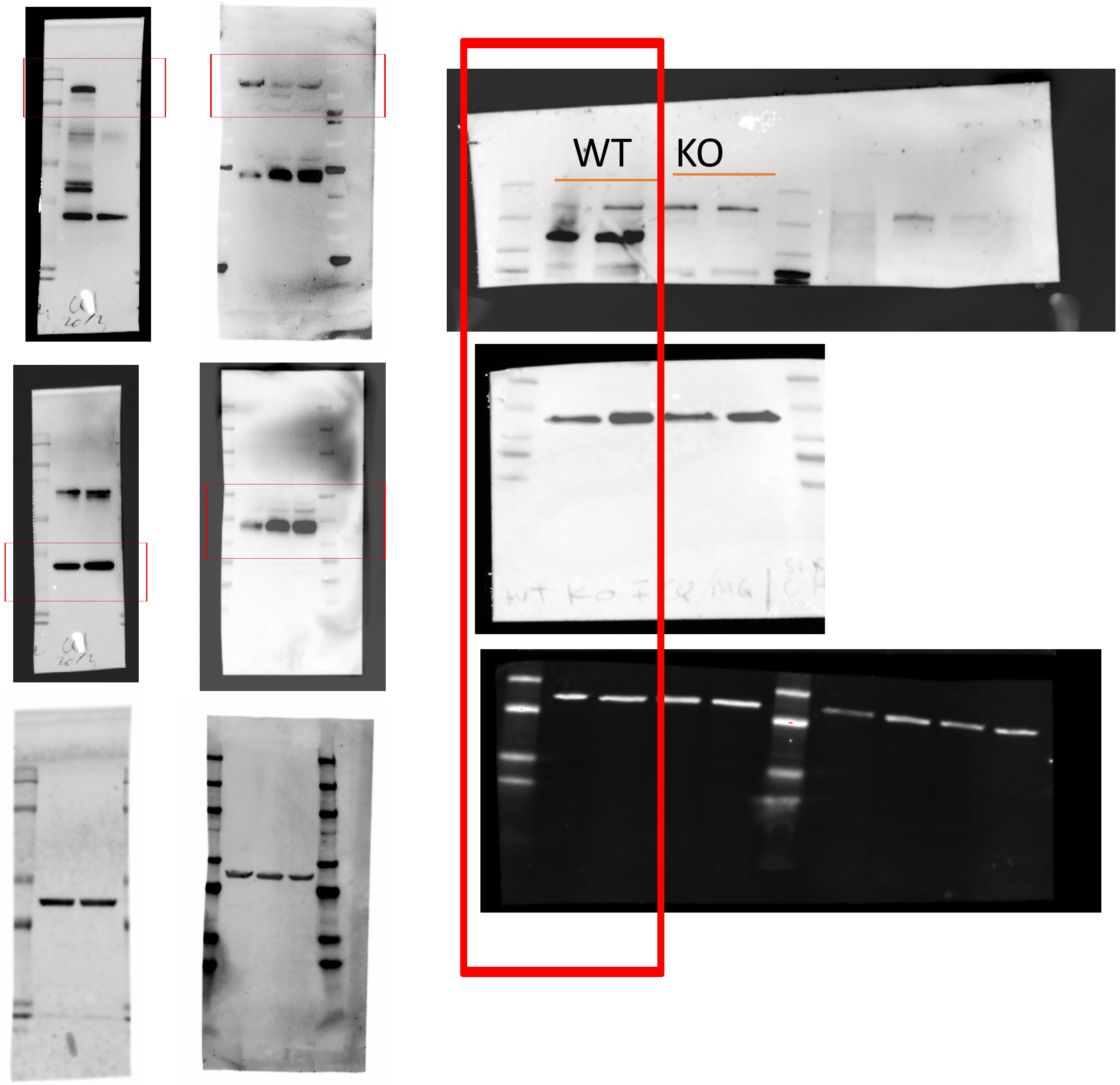

Figure 2 blots

GFP

Myc

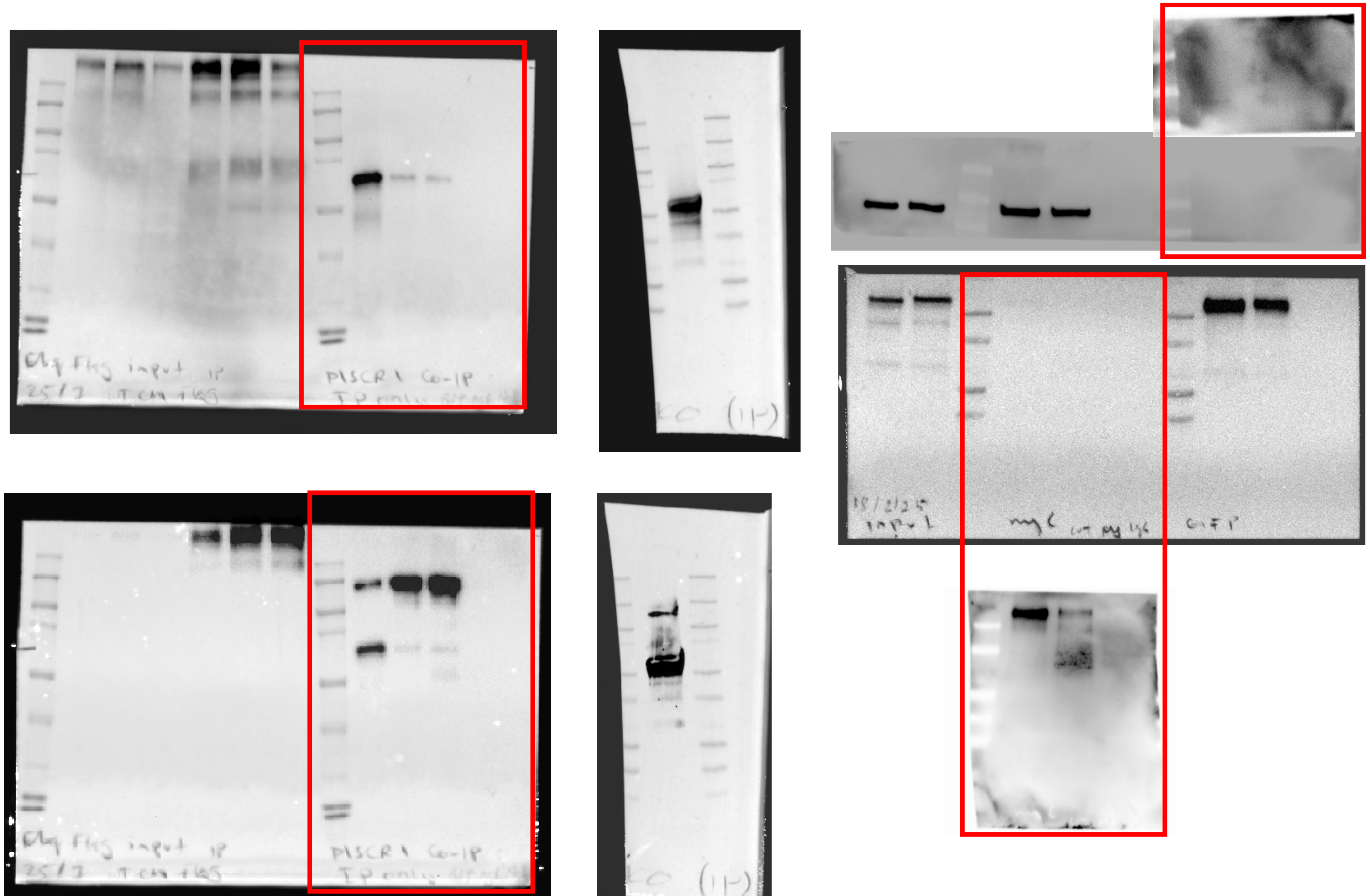

Figure 2 blots (Cont)

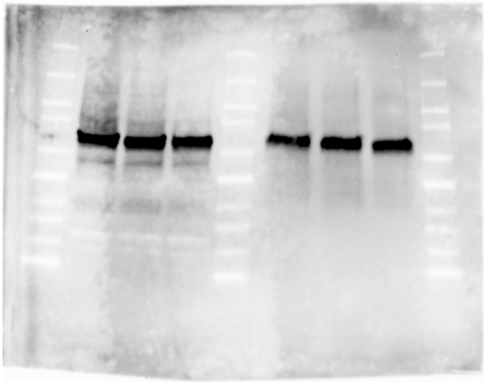

GFP

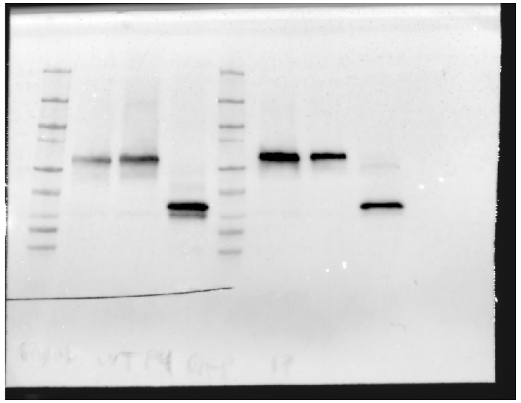

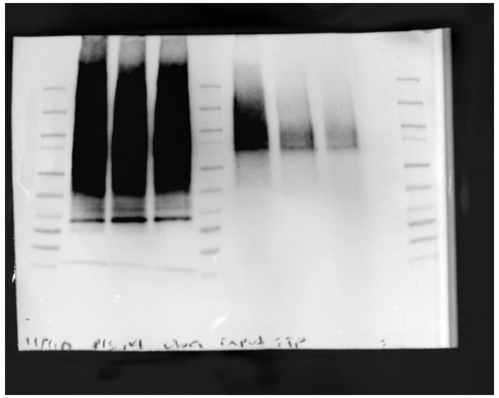

HA

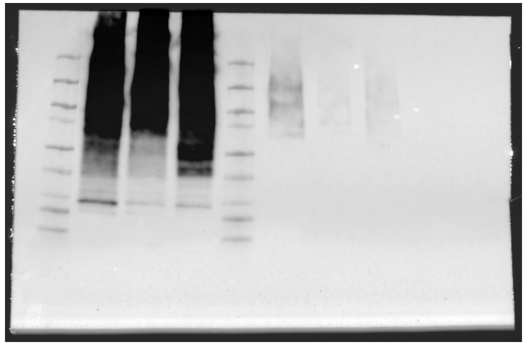

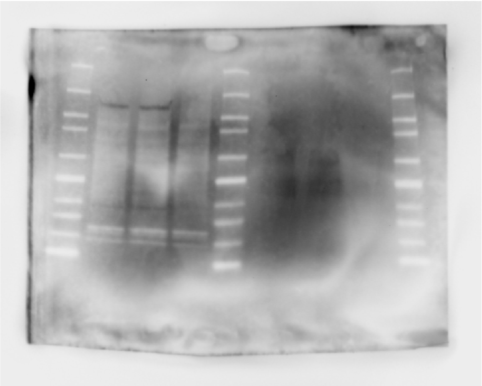

Myc

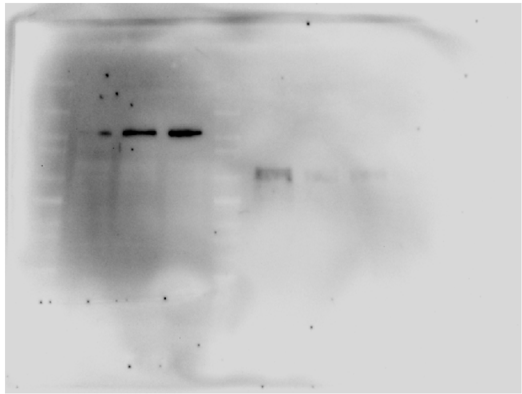

Figure 3 blots

NEDD4-2

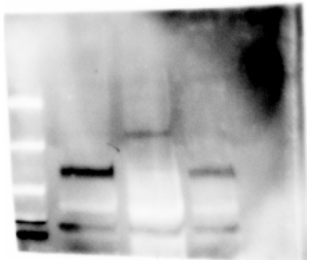

PLSCR1

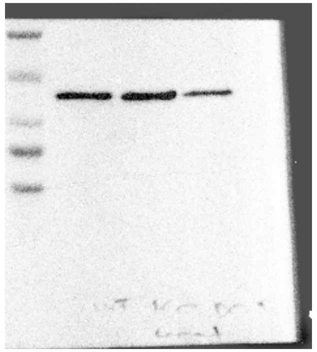

actin

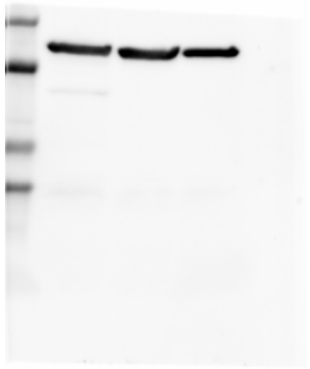

Figure 4 blots

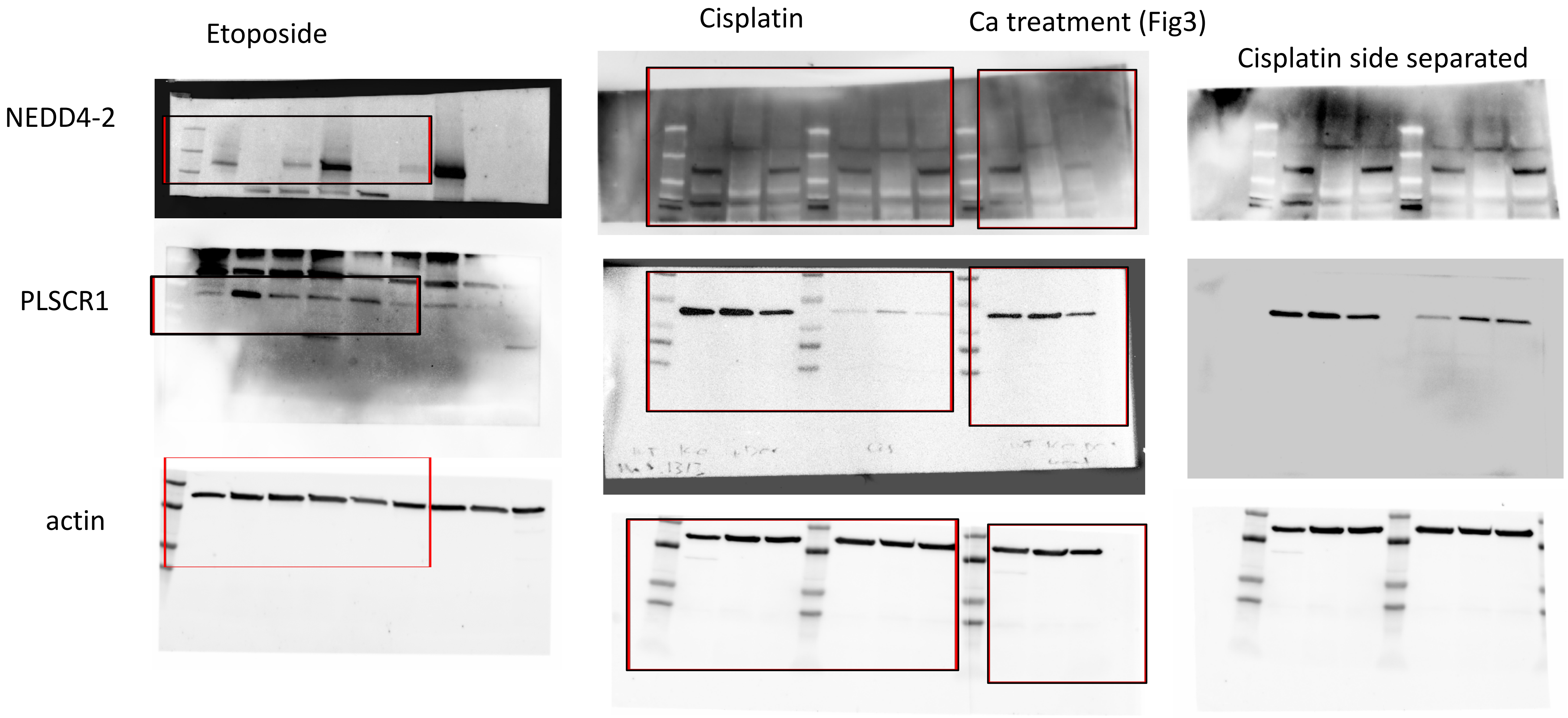

Figure 6 blots

NEDD4-2

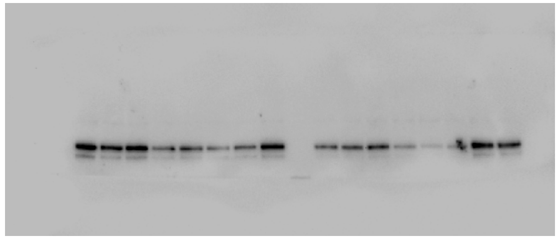

PLSCR1

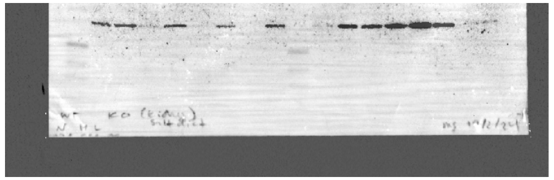

GAPDH

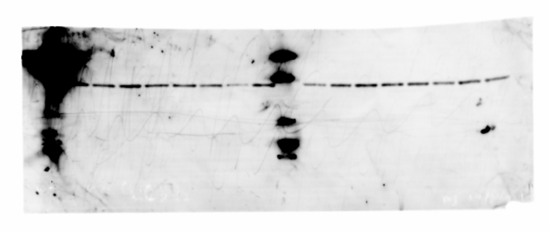

Stain free blot

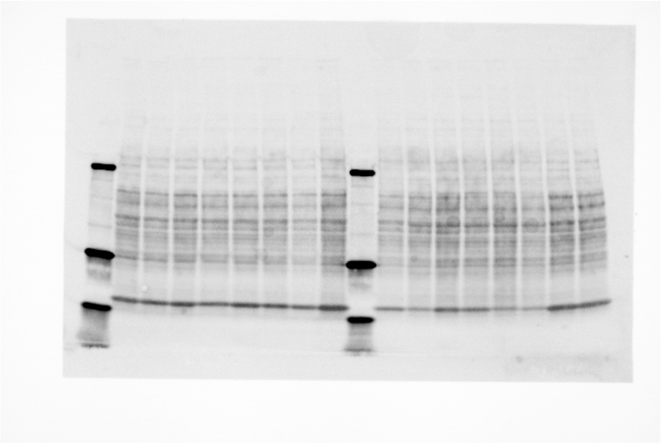

Supplement: Supplementary file 2 — Uncropped Immunoblots [file 41420_2025_2700_MOESM2_ESM.pdf]
